# Metastasis from scapular Ewing’s sarcoma presenting as sutural diastasis: An unusual presentation

**DOI:** 10.4103/0973-6042.68415

**Published:** 2010

**Authors:** Naiyer Asif, Abdul Qayyum Khan, Yasir Salam Siddiqui, Hamid Mustafa

**Affiliations:** Department of Orthopaedic Surgery, J. N. Medical College, A.M.U., Aligarh, India; 1 Department of Radiotherapy, J. N. Medical College, A.M.U., Aligarh, India

**Keywords:** Ewing’s sarcoma, non-osteogenic, skull vault, sutural diastasis

## Abstract

Ewing’s sarcoma is a malignant non-osteogenic primary tumor of the bone. It is one of the most common primary malignant tumors of bone. Peak incidence is noticed in second decade of life with male preponderance of 1.6:1. It occurs most frequently in long bones and flat bones of pelvic girdles. In 30% cases, Ewing’s sarcoma is multicentric in origin. In 14-50%, multiple metastases are present at the time of diagnosis. CNS spread is rare and isolated CNS involvement is not seen. Skull metastasis of Ewing’s sarcoma is not rare compared to primary Ewing’s sarcoma of the skull, but the actual frequency is unknown. We wish to report a case of “Primary Ewing’s sarcoma of scapula with metastasis to Skull Vault in a Child resulting in sutural diastasis” diagnosed by clinicoradiological examination and confirmed by histopathology.

## INTRODUCTION

Ewing’s sarcoma is a malignant non-osteogenic primary tumor of the bone. It is one of the most common primary malignant tumors of bone. Peak incidence is noticed in second decade of life with male preponderance of 1.6:1. It occurs most frequently in long bones and flat bones of pelvic girdles.[1] In 30% cases, Ewing’s sarcoma is multicentric in origin.[2] In 14-50%, multiple metastases are present at the time of diagnosis.[3] CNS spread is rare and isolated CNS involvement is not seen.[4] Skull metastasis of Ewing’s sarcoma is not rare compared to primary Ewing’s sarcoma of the skull, but the actual frequency is unknown.[13–15] We wish to report a case of “Primary Ewing’s sarcoma of scapula with metastasis to Skull Vault in a Child resulting in sutural diastasis” diagnosed by clinicoradiological examination and confirmed by histopathology.

Ewing’s sarcoma is a malignant non-osteogenic primary tumor of the bone. Originally, James Ewing described it in 1921 as a tumor arising from undifferentiated osseous mesenchymal cells; however, recent studies suggest that Ewing’s tumor may be neuroectodermally derived from the primitive neural tissue.[8] Ewing’s sarcoma is a malignant tumor of the bone that usually occurs during the first two decades of life. It has predilection for the trunk and long bones. 30% of these tumors are multicentric and metastasis is often present at the time of diagnosis.[2,3] Primary Ewing’s sarcoma of skull vault is very rare. Skull metastasis of Ewing’s sarcoma is not rare compared to primary Ewing’s sarcoma of the skull, but the actual frequency is unknown. The location of skull metastasis of Ewing’s sarcoma is also not clear.[13–15] The earliest symptom is pain. Other common symptoms include fever, anemia, and non-specific signs of inflammation, such as an increase in sedimentation rate, moderate leukocytosis, and an increase in serum LDH. Classical radiological presentation of Ewing’s sarcoma is a destructive lesion in the diaphysis of long bones with an onionskin periosteal reaction. Skip metastases are rare in Ewing’s sarcoma. They are a well-recognized feature of osteosarcoma and are associated with a poorer prognosis than cases clear of such lesions. Skip lesions appear to be rare in Ewing’s sarcoma,[16–19] and the prognostic implications of skip lesions in Ewing’s sarcoma remains unclear. In flat bones, Ewing’s sarcoma appears as a nonspecific destructive lesion. From remote primary sites like pelvis and long bones, Ewing’s sarcoma may metastasize to skull. Secondry skull lesions presents with osteolysis with erosion of inner and outer table associated with soft tissue swelling on plain radiographs.

The management of Ewing’s sarcoma includes multiagent neoadjuvent chemotherapy followed by enblock excision of the tumor mass. Postoperative radiotherapy is given if there is doubt of tumor residua. The most unfavorable prognostic factor in Ewing’s sarcoma is the presence of distant metastasis at diagnosis. Other unfavorable prognostic factors include an age older than 10 years, a size larger than 200 ml, more central lesions (as in the pelvis or spine), and poor response to chemotherapy.[5–7]

## CASE REPORT

**Figure 1:**
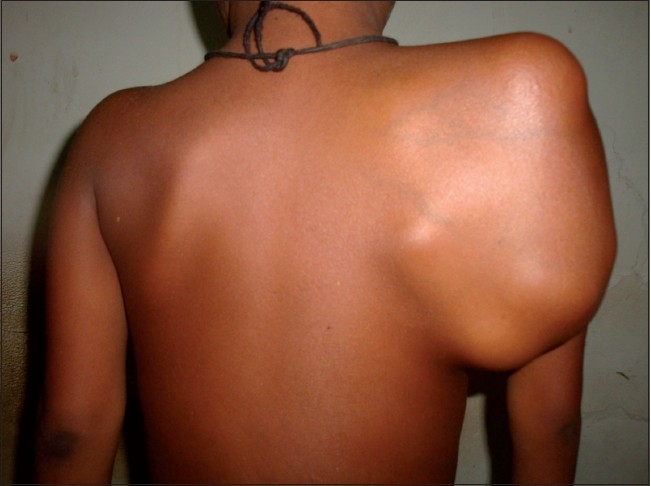
Clinical photograph showing massive scapular swelling

**Figure 2:**
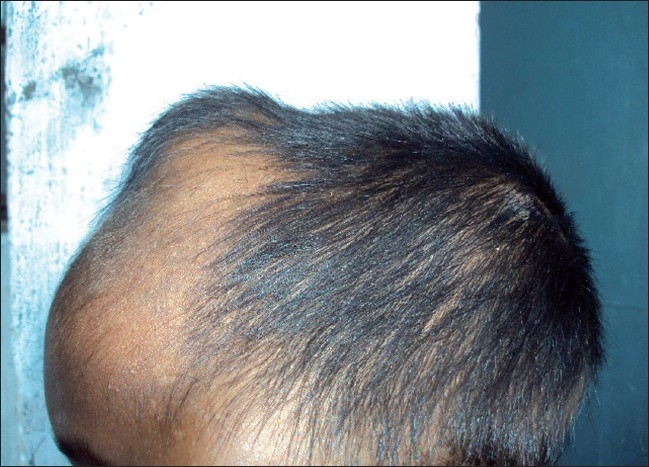
Clinical photograph showing skull vault swelling in the frontoparietal region

**Figure 3:**
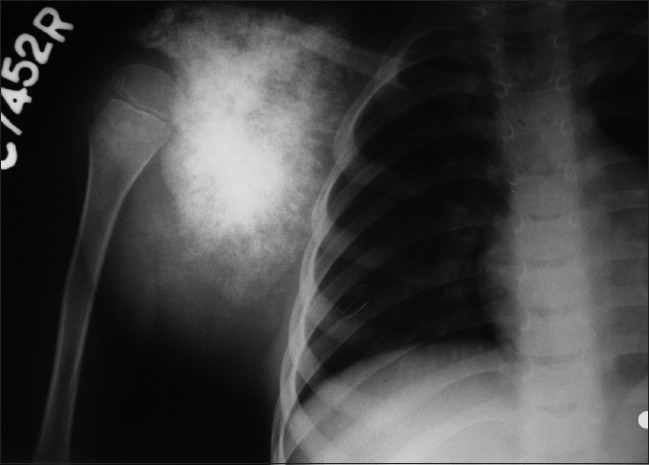
Pre-treatment radiograph of right shoulder showing a sclerotic lesion of scapula with loss of definition of margins of scapula and soft tissue mass

**Figure 4:**
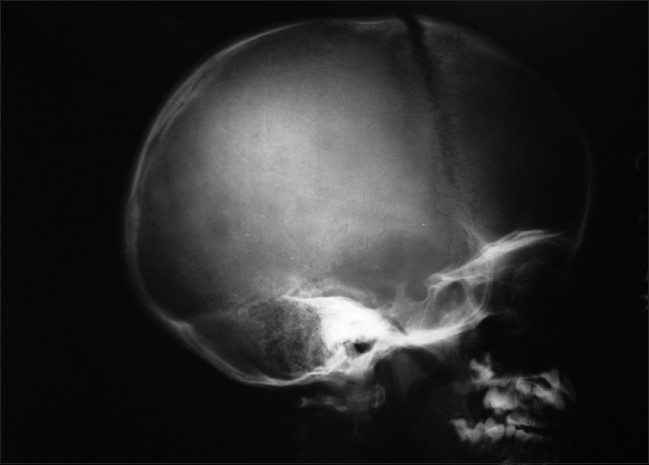
Lateral radiograph of skull vault showing an osteolytic lesion with erosion of inner and outer tables associated with sutural diastasis and periosteal reaction

**Figure 5:**
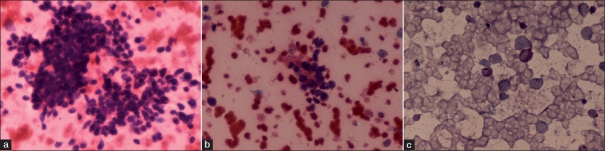
FNAC from scapular mass was consistent with small round cell tumor of childhood – Ewing’s sarcoma. (a) Photomicrograph showing loose clusters and the discrete cell population of monomorphic, round cells with scant cytoplasm, slightly irregular nuclei, and occasional pseudo-rosette and focal necrosis. Note that a mixture of two types of cell population – small and large – can be appreciated, one of the criteria for diagnosis of Ewing’s sarcoma (H and E stain). (b) Photomicrograph focused to show rosette like structure formation, as occasionally seen in cases of Ewing’s sarcoma (H and E stain). (c) Photomicrograph showing PAS positivity in few viable cells (PAS stain)

**Figure 6:**
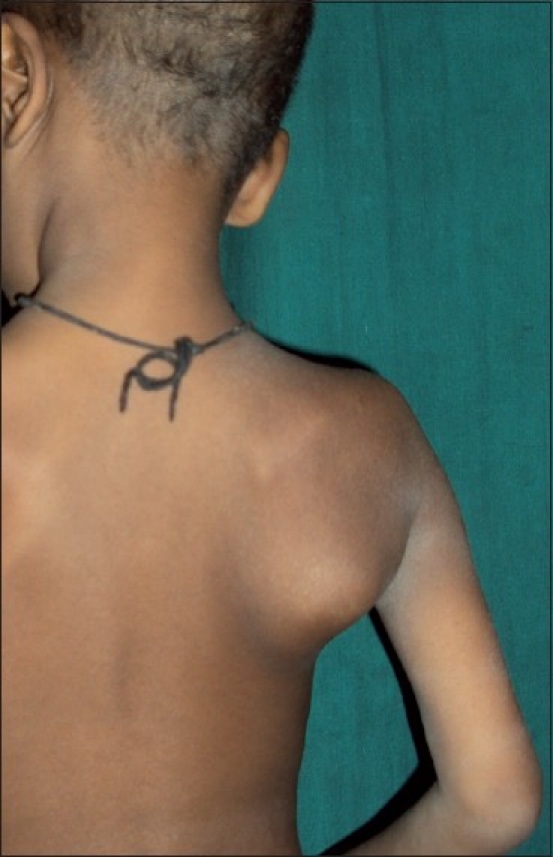
Clinical photograph showing significantly reduced scapular swelling (post treatment)

**Figure 7:**
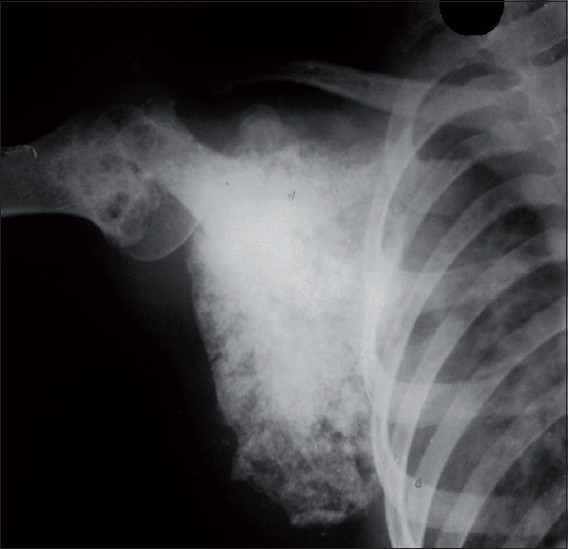
Radiograph of right shoulder showing extensive sclerosis of the lesion after chemoradiation

**Figure 8:**
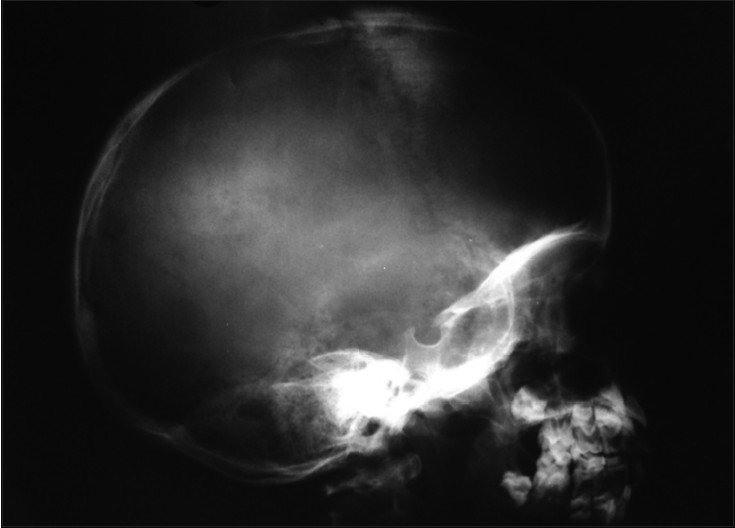
Post-treatment lateral radiograph of skull vault showing sclerosis in and around the lesion. The sclerosis seems to be bridging the diastasis

A 5-year-old boy presented with 5 months history of pain followed by swelling around the right shoulder girdle. 2 months prior to consultation at J N Medical College, AMU, Aligarh, patient’s mother noticed gradually increasing swelling over scalp. The patient had no history of headache, weakness of upper and lower limbs. There were no signs of raised intracranial tension. There was no evidence of weakness of upper and lower limbs and other localizing signs of intracranial mass. On clinical examination, the shoulder girdle swelling was diffuse with ill-defined margins, firm to hard in consistency with tense and shiny over lying skin [Figure 1]. Scalp swelling was present in frontoparietal region, which was hard in consistency and tender [Figure 2]. Rest of the skeletal survey was normal. At the time of presentation, plain radiograph of the affected shoulder showed a sclerotic lesion of scapula with loss of definition of margins of scapula with soft tissue mass [Figure 3]. Lateral radiograph of skull showed osteolysis with erosion of inner and outer table associated with sutural diastasis and periosteal reaction [Figure 4]. All the blood parameters were normal except the alkaline phosphatase level, which was raised. FNAC from the scapular and scalp swelling was consistent with malignant small round cell tumor - Ewing’s sarcoma [Figure 5]. The patient was subjected to local radiotherapy followed by multiagent chemotherapy after histological confirmation of Ewing’s sarcoma. The patient showed dramatic response to chemoradiation therapy both clinically and radiologically [Figures 6 and 7]. The post-treatment lateral radiograph of skull showed development of sclerosis in and around the lesion [Figure 8]. The sclerosis seems to be bridging the diastasis.

## DISCUSSION

Ewing sarcoma is the most common primary bone tumor of the flat bones. Primary Ewing’s sarcoma of skull vault is very rare. Skull metastasis of Ewing’s sarcoma is not rare compared to primary Ewing’s sarcoma of the skull, but the actual frequency is unknown.[13–15]

In our patient appearance of a scapular lesion was consistent with osteogenic sarcoma, as the lesion is primarily sclerotic and it does not look like Ewing’s at all. Radiographic appearance of Ewing’s sarcoma in the thorax is quite variable and the lesion may be primarily lytic or sclerotic or may represent the mixture of the two.[10,11] A universal feature of the disease is the presence of a large soft tissue mass relatively larger than the extent of bone changes as was evident in our patient. Sometimes the only apparent change in a radiograph is the presence of a mass, cortical erosion or periosteal reaction being hardly visible.[12] Unlike osteogenic sarcoma, Ewing’s sarcoma occurs almost as frequently in flat bone as in long bones, with a small bias toward long bones. As Ewing’s sarcoma shows close resemblance to changes seen in osteomyelitis, osteogenic sarcoma, eosinophilic granuloma, metastatic deposits, Vohkam VG *et al*, concluded that diagnosis of Ewing’s sarcoma by roentgenography alone is in some cases not warranted at all and in others it is, at best, a presumptive diagnosis which requires confirmation by histological examination in all.[12]

Secondry skull lesions presents with osteolysis with erosion of inner and outer table associated with soft tissue swelling on plain radiographs. In our patient metastasis from primary lesion resulted in sutural diastasis. Similar skull involvement may also be seen in lesions in Eosinophilic granuloma/Hand–Schuller–Christian syndrome, Burkitt’s lymphoma, fibrous dysplasia, aneurysmal bone cysts, osteoclastoma, and giant cell reparative granuloma. Radiologically it is difficult to distinguish them; however, they can be distinguished by a different pattern of destruction of skull vault, presence of soft tissue swelling, calcification, cystic component, and septations. However, histopathology is more specific for their differentiation. MRI is the imaging modality of choice for evaluating the extent of the primary tumor, to monitor the response to neoadjuvent chemotherapy and to follow up unresected Ewing’s sarcoma. Bone scintigraphy is necessary to detect skeletal metastasis at the earliest, and 201-thalium scanning has been shown to be sensitive in the monitoring of the response[9] to treatment. Our patient had evidence of distant (skull) metastasis at the time of diagnosis, an axial not an appendicular lesion and extensive lesion–all unfavorable prognostic factors for disease.

## CONCLUSION

Skull metastasis of Ewing’s sarcoma is not rare compared to primary Ewing’s sarcoma of the skull, but metastasis resulting in sutural diastasis is rare. Patient presents with scalp swelling with or without non-specific neurological signs. CT scan and MRI delineate skull vault lesion with associated epidural soft tissue mass. Histopathology differentiates it from other primary skull vault lesions.
